# Sine-Cosine-Adopted African Vultures Optimization with Ensemble Autoencoder-Based Intrusion Detection for Cybersecurity in CPS Environment

**DOI:** 10.3390/s23104804

**Published:** 2023-05-16

**Authors:** Latifah Almuqren, Fuad Al-Mutiri, Mashael Maashi, Heba Mohsen, Anwer Mustafa Hilal, Mohamed Ibrahim Alsaid, Suhanda Drar, Sitelbanat Abdelbagi

**Affiliations:** 1 Department of Information Systems, College of Computer and Information Sciences, Princess Nourah Bint Abdulrahman University, P.O. Box 84428, Riyadh 11671, Saudi Arabia; 2 Department of Mathematics, Faculty of Sciences and Arts, King Khalid University, Muhayil Asir 63311, Saudi Arabia; 3 Department of Software Engineering, College of Computer and Information Sciences, King Saud University, P.O. Box 103786, Riyadh 11543, Saudi Arabia; 4 Department of Computer Science, Faculty of Computers and Information Technology, Future University in Egypt, New Cairo 11835, Egypt; 5 Department of Computer and Self Development, Preparatory Year Deanship, Prince Sattam Bin Abdulaziz University, AlKharj 11942, Saudi Arabia

**Keywords:** cyber-physical systems, feature selection, deep learning, metaheuristics, cybersecurity

## Abstract

A Cyber-Physical System (CPS) is a network of cyber and physical elements that interact with each other. In recent years, there has been a drastic increase in the utilization of CPSs, which makes their security a challenging problem to address. Intrusion Detection Systems (IDSs) have been used for the detection of intrusions in networks. Recent advancements in the fields of Deep Learning (DL) and Artificial Intelligence (AI) have allowed the development of robust IDS models for the CPS environment. On the other hand, metaheuristic algorithms are used as feature selection models to mitigate the curse of dimensionality. In this background, the current study presents a Sine-Cosine-Adopted African Vultures Optimization with Ensemble Autoencoder-based Intrusion Detection (SCAVO-EAEID) technique to provide cybersecurity in CPS environments. The proposed SCAVO-EAEID algorithm focuses mainly on the identification of intrusions in the CPS platform via Feature Selection (FS) and DL modeling. At the primary level, the SCAVO-EAEID technique employs Z-score normalization as a preprocessing step. In addition, the SCAVO-based Feature Selection (SCAVO-FS) method is derived to elect the optimal feature subsets. An ensemble Deep-Learning-based Long Short-Term Memory–Auto Encoder (LSTM-AE) model is employed for the IDS. Finally, the Root Means Square Propagation (RMSProp) optimizer is used for hyperparameter tuning of the LSTM-AE technique. To demonstrate the remarkable performance of the proposed SCAVO-EAEID technique, the authors used benchmark datasets. The experimental outcomes confirmed the significant performance of the proposed SCAVO-EAEID technique over other approaches with a maximum accuracy of 99.20%.

## 1. Introduction

A Cyber-Physical System (CPS) is a type of computing system combined with physical gadgets and can be broadly utilized in various areas, namely, energy, manufacturing, safety management, and traffic control [1]. The most common enabler for the intelligence sector is the integration of the Cloud Computing (CC) technique and CPS, which remains a common trend with numerous real-time cases, for example, Small and Medium Enterprises (SMEs), supporting industrial cluster collaboration with business cooperation and cloud manufacturing service platforms [2]. With the help of CC, more optimized techniques are constituted to enrich the robustness and reliability of the system and collaborate with other systems in order to enlarge the efficiency of the functions at limited data usage for CPSs. Although Information and Communication Technology (ICT) is an advanced part of CPSs, cybersecurity is considered to be a challenging issue in many domains. Intrusion is one of the primary complications encountered in CPSs [3]. In the past, special attention has been paid to the development of secure CPSs. Further, efforts have been taken to maximize the integrity of CPSs with Intrusion Detection (ID), which has become a predominant application. Generally, an Intrusion Detection System (IDS) is used to prevent attacks in an efficient manner [4]. An IDS is referred to as a tool that detects or classifies cyberattacks in a network or host by implementing some identification approaches. There are two classes present in IDSs, such as Anomaly-based IDSs and Signature-based IDSs (AIDSs and SIDSs). In SIDSs, attacks are identified with regard to the predefined sign or pattern of attacks. In AIDS networks, traffic patterns are monitored and compared against regular or normal paradigms in the network so as to identify intrusions [5]. 

Several AI-based IDS approaches have been devised so far to ensure the security of CPSs. In spite of the fact that the presented methods show good performance, they are grounded on the assumption that the dataset reflects the real-time situations of cyberattacks [6]. However, on a real-time basis, users access datasets with limited examples of cyberattacks. As cyberattacks evolve in terms of complexity and volume, Machine Learning (ML) techniques have been applied to manage various malicious performance and cybersecurity attacks. The CPS unites the calculation with that of the physical process [7]. The embedded network computer controls and monitors the physical processes, normally with feedback loops in which the physical processes affect the computation simultaneously. In general, ML techniques are prone to data pollution attacks. Therefore, it is important to enhance network security and achieve a strong ML-based network method in the development of CPSs [8]. ML approaches are broadly leveraged in the detection of cyber intrusions due to their timely and automatic manner of action. The opportunity to make an adaptable and scalable detection system is offered by DL methods. The DL method is utilized with unsupervised and supervised techniques [9,10]. Unsupervised techniques are used to make labels for non-labeled samples.

The current study presents a Sine-Cosine-Adopted African Vultures Optimization with Ensemble Autoencoder-based Intrusion Detection (SCAVO-EAEID) technique for cybersecurity in the CPS environment. At the primary level, the proposed SCAVO-EAEID technique employs the Z-score normalization process as a preprocessing step. Then, the SCAVO-based Feature Selection (SCAVO-FS) method is applied to elect the optimal feature subsets. This step shows the novelty of the work. For intrusion detection, the ensemble Deep-Learning-based Long Short-Term Memory–Auto Encoder (LSTM-AE) model is employed. Finally, the Root Mean Square Propagation (RMSProp) optimizer is used for the hyperparameter tuning of the LSTM-AE model. To demonstrate the remarkable performance of the proposed SCAVO-EAEID technique, benchmark datasets were used. In short, the key contributions of the current study are summarized herewith.

- An automated SCAVO-EAEID technique comprising Z-score normalization, the SCAVO-FS technique, LSTM-AE-based intrusion detection, and the RMSProp optimizer is developed for intrusion detection in the CPS environment. To the best of the researchers’ knowledge, no researchers have proposed the SCAVO-EAEID technique in the literature.
- A new SCAVO-FS technique has been designed by integrating the sine-cosine scaling factor and the AVO algorithm for the repositioning of the vultures at the end of the iterations.
- Both the RMSProp optimizer and the LSTM-AE model are employed in this study for the intrusion detection process.
- The performance of the proposed SCAVO-EAEID technique was validated using two benchmark datasets such as the NSL-KDD 2015 and CICIDS2017 datasets.

The rest of the paper is organized as follows. Section 2 discusses the related works, and Section 3 offers the proposed model. Then, Section 4 provides the analytical results, and Section 5 concludes the paper.

## 2. Related Works

Huang et al. [11] introduced a new federated Execution and Evaluation dual network model (EEFED), which allows different federal participants to identify the local detection model. This phenomenon undermines the primary objective of Federated Learning (FL). Mansour [12] proposed a novel Poor and Rich Optimization with the DL method for BC-Assisted IDS in CPS Environments (PRO-DLBIDCPS). At first, the model implemented the Adaptive Harmony Search Algorithm (AHSA)-based FS method for an appropriate selection of the feature subset. The PRO technique with the Attention-based Bi-Directional Gated RNN (ABi-GRNN) system was used in this study for both the detection and classification of the intrusions. Henry et al. [13] introduced a method integrating CNN and GRU in which both combinations were used for the optimization of the network parameters. In this work, the author utilized the CICIDS-2017 benchmark datasets. 

Ortega-Fernandez et al. [14] introduced the Network IDS (NIDS) model based on the DAE, trained with network flow data. This model had a benefit, i.e., no need to have previous knowledge about the underlying architecture or the network topology. The experimental result showed that the presented method was capable of detecting the anomalies, caused by distributed DoS attacks. The proposed method provided a low false alarm rate and high detection accuracy. It also outperformed the other methods and acted as a baseline and a state-of-the-art model for the unsupervised learning model. Likewise, the DAE model is capable of detecting abnormal behaviors in legitimate devices after an attack. Wang et al. [15] introduced a knowledge distillation method-based Triplet CNN technique to heavily enhance the speed and improve the AD performance of the model for industrial CPS in addition to the reduction of model complexity. In particular, during the training, a robust model loss function was devised to enhance the network’s stability. A novel NN training model named K-fold cross-training was developed in this study to enhance anomaly detection performance. 

Mittal et al. [16] introduced a new technique for the IDS. The presented technique applied a new variant of the gravitational search technique to attain the optimum cluster. In the presented method, Kbest was adapted as an exponentially-declining function with logistic-mapping-based chaotic behaviors. Presekal et al. [17] developed a new technique for online cyberattack awareness. This technique improves the power grid resilience and assists the power system operators during localization and identification processes of the active attack locations in the Operational Technology (OT) network on a real-time basis. The presented technique used a hybrid DL mechanism, i.e., deep convolutional network with Graph Convolutional LSTM (GC-LSTM), for time-series classification-related AD.

Though several ML and DL models have been proposed earlier for intrusion detection and classification, a need still exists to enhance the classification performance. Owing to the continuous evolution of the model, the number of parameters in DL models also increases quickly, bringing model overfitting issues. Since the trial-and-error method is a tedious and erroneous process for hyperparameter tuning, the metaheuristic algorithms are applied. Therefore, in this work, the authors employ the BO algorithm for the parameter selection of the LSTM-AE model.

## 3. Proposed Model

**Figure 1:**
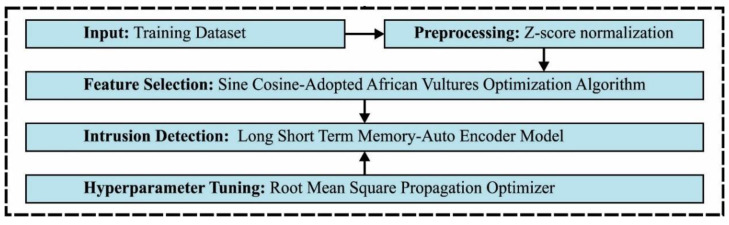
Working Principle of the SCAVO-EAEID technique.

In this study, a new SCAVO-EAEID method has been developed for the classification of intrusions in the CPS environment. The SCAVO-EAEID algorithm performs the detection of intrusions in the CPS environment using major sub-processes namely, the LSTM-AE-based classification, Z-score normalization, SCAVO-FS technique, and the RMSProp optimizer-based hyperparameter tuning. The workflow of the proposed model is demonstrated in Figure 1.

### 3.1. Data Used

In the current research work, the proposed model was experimentally validated upon two benchmark datasets such as the NSLKDD2015 (https://www.unb.ca/cic/datasets/nsl.html, accessed on 12 February 2023) and CICIDS2017 datasets (https://www.unb.ca/cic/datasets/ids-2017.html, accessed on 12 February 2023). The NSL-KDD 2015 dataset has a total of 125,973 samples with 41 features. The NSL-KDD 2015 dataset does not include any redundant records in the training set. So, the classifiers remain unbiased towards more frequent records. Likewise, CICIDS2017 has a total of 2,830,743 samples with 80 features. The CICIDS2017 dataset contains both benign and the most up-to-date common attacks, which resemble the true real-world data (PCAPs). It also includes the network traffic analysis results with the help of CICFlowMeter and labeled flows based on a few parameters such as the time stamp, source, destination IPs, source and destination ports, protocols, and attack.

### 3.2. Data Preprocessing

At first, the SCAVO-EAEID technique applies the Z-score normalization as a preprocessing step. This procedure is leveraged to attain the normalized values or ranges of the input dataset, from the unstructured dataset, by applying the concepts such as mean and standard deviation [18]. The normalized values or ranges can be obtained by dividing the presented data of every gate using standard deviation values and then subtracting the mean of every gate from that value. Equations (1) and (2) are utilized to map the value of the new input between the target ranges, i.e., [x,y].

(1) $$A_{Za(t)}=\frac{A\left(t\right)-A_{j}\left(t\right)}{A_{j}(t)}$$

(2) $$A_{Zad(t)}=c+\frac{\left(d-c)(A_{ZS}(t)-A_{ZSmin}\right)}{A_{ZSmax}-A_{ZSmin}}$$

Here, $A_{Zad(t)}$ denotes the scaling value of the z-score-normalized field $A_{Za(t)}.A_{i}(t)$ and $A_{j}(t)$ are evaluated from the training datasets, which represent the standard deviation and mean at every gate time t, whereas $A_{ZS_{min}}$ and $A_{ZSmax}$ denote the absolute initial and final gate values that are noticed for the gate period during the testing dataset.

### 3.3. Processes Involved in the SCAVO-FS Technique

In this work, the SCAVO-FS system is derived to elect the optimal feature subsets. The AVO algorithm is stimulated by the navigational and foraging behaviors of the African vultures [19]. The biological nature of these vultures with regard to competing and searching for food is outlined in four different phases as follows. Consider N number of vultures in the atmosphere, which describes the amount of population, i.e., n={1,2,…,N}. In the following equation, the fitness function of every location is evaluated.

(3) $$p_{n}=\frac{F_{n}}{\sum_{n=1}^{N}F_{n}}$$

Here, $p_{n}$ represents the probability of choosing either the first or second group, $F_{n}$ denotes the fitness function of the nth location [19]. Next, the formation of the 1st and 2nd groups during all the iterations is attained as follows.

(4) $$R(it)=\left\{\begin{array}{l} first\;group,p_{n}=L_{1}\\ second\;group,p_{n}=L_{2} \end{array}\right.$$

Here, the ranges of $L_{1}$ and $L_{2}$ are $0\leq L_{1},L_{2}\leq 1$ and $L_{1}+L_{2}=1$, respectively. The satiated vulture with sufficient energy can move a long distance to find food, whereas a hungry one cannot fly longer as denoted below.

(5) $$A=(2\times rand_{1}+1)\times x\times (1-\frac{it}{IT_{max}})+y$$

(6) $$y=h\times ({sin}^{z}(\frac{\Pi }{2}\times \frac{it}{IT_{max}})+cos(\frac{\Pi }{2}\times \frac{it}{IT_{max}})-1)$$

In Equations (5) and (6), A denotes the vulture with high energy, it and $IT_{max}$ denote the present and the maximal iterations, correspondingly, h and $rand_{1}$ indicate the random number in the range of $\left[-1,1\right],\left[-2,2\right]$, and [0,1], correspondingly; and z describes the probability of entering the exploration phase.

The procedure of seeking food by African vultures defines the exploration stage in which the parameter $p_{1},0\leq p_{1}\leq 1$ defines the selection of strategy.

(7) $$P(it+1)=\left\{\begin{array}{l} (6),p_{1}\geq rand_{2}\\ (8),p_{1}<rand_{2} \end{array}\right.$$

(8) P(it+1)=R(it)−D(it)×A

(9) D(it)=|q×R(it)−P(it)|

In this expression, P(it+l) represents the location vector of the vulture in the second iteration. $q=2\times rand_{3}$, where $rand_{3}$ denotes the randomly generated value in the range of [0,1].

(10) $$P\left(it+1\right)=R\left(it\right)-A+rand_{4}\times \left(\left(u_{b}-1_{b}\right)\times rand_{5}+1_{b}\right)$$

$u_{b}$ and $l_{b}$ denote the upper and lower boundaries correspondingly, while $Rand_{4}$ and $Rand_{5}$ indicate the randomly generated integers that lie in the range of 0 to 1. 

The exploitation phase includes two stages with dissimilar strategies. The selection of any method relies on both $p_{2}$ and $p_{3}$ parameters, while its values lie in the range of 0 to 1. If |F| ranges between 0.5 and 1, then the exploitation phase enters the initial phase, which defines the siege fight and rotating flight strategies.

(11) $$P(it+1)=\left\{\begin{array}{l} (10),p_{2}\geq rand_{6}\\ (11),p_{2}<rand_{6} \end{array}\right.$$

Here, $rand_{6}$ denotes a randomly generated value in the range of [0, 1]. The solution to Equation (11) is given below.

(12) $$P(it+l)=D(it)\times (A+rand_{7})-d(it)$$

(13) D(it)=R(it)−P(it)

Next, the rotational flight of the vulture can be modeled as given below.

(14) $$P(it+l)=R(it)-(M_{1}+M_{2})$$

(15) $$M_{1}=R(it)\times (\frac{rand_{8}\times P(it)}{2\pi })\times cos(P(it))$$

(16) $$M_{2}=R(it)\times (\frac{rand_{9}\times P(it)}{2\pi })\times sin(P(it))$$

Here, $rand_{8}$ and $rand_{9}$ denote the two randomly-generated integers in the range of [0, 1]. If $\left|F\right|>0.5,$ then the exploitation phase enters the second phase, which describes the aggressive siege and accumulation fight strategies for finding the food. Based on the following condition, any strategy can be selected.

(17) $$P(it+1)=\left\{\begin{array}{l} (16),p_{3}\geq rand_{7}\\ (19),p_{3}<rand_{7} \end{array}\right.$$

where

(18) $$P(it+1)=\frac{B_{1}+B_{2}}{2}$$

(19) $$B_{1}=Best_{vulture1}(it)-\frac{Best_{vulture1}(it)\times P(it)}{Best_{vulture1}(it)\times P(it{)}^{2}}\times A$$

(20) $$B_{2}=Best_{vulture2}(it)-\frac{Best_{vulture2}(it)\times P(it)}{Best_{vulture2}(it)\times P(it{)}^{2}}\times A$$

$Best_{vulture1}$ (it) and $Best_{vulture2}$ (it) represent the better vultures of the first and second groups, correspondingly as shown below.

(21) P(it+1)=R(it)−|d(it)|×A×Levy(d)

Here, $d_{1}$ signifies the dimension of the problem. *Levy*($d_{1}$) is evaluated as given below.

(22) $$\mathrm{Levy}(x)=0.01\times \frac{u\times \sigma }{|v{|}^{1/\beta }},\sigma =(\frac{Y(1+\beta )\times sin(\frac{\pi \beta }{2})}{Y(1+2\beta )\times \beta \times 2(\frac{\beta -1}{2})}{)}^{1/\beta }$$

The best possible solution is not known at the initial stage of the AVO algorithm. Therefore, it is better to use a large step in the beginning, which might generate the calculation process farther from the optimum location [20]. Consequently, the scaling variable is used for changing the situation in the initial phase. In the SCAVO algorithm, the vulture is repositioned at the end of every iteration based on sine- and cosine-adapted scaling factors, as given below [20].

(23) $$P(it+1{)}_{New}=P(it+1)\times SCaSF$$

Now, the *SCaSF* denotes the scaling factor as follows

(24) $$SCaSF=\left\{\begin{array}{cc} sin\left(W_{1}-W_{2}\frac{it}{{Max}_{-}it}\right) & if\;RNDI<0.5\\ \cos\left(W_{1}-W_{2}\frac{it}{{Max}_{-}it}\right) & if\;RNDI\geq 0.5 \end{array}\right.$$

In Equation (24), *RNDI* indicates a randomly-generated value; $W_{1}$ and $W_{2}$ denote the weighting factors, and it and Max_it indicate the current and maximal iteration, respectively. The scaling factor is used to control the vulture’s development at the initial stage. Different upsides of W are tried and appointed for a proper choice of $W_{1}$ and $W_{2}$. It is to be noted that the best possible results are attained when $W_{1}$ and $W_{2}$ are selected as 10 and 9 correspondingly. The scaling factor has changed the vulture’s position at the underlying period of the pursuit interaction, thus increasing the hunting capability of the AVO technique further. The calculation should provide the option to locate the best location between two locations for the exploitation of the search range. These cycles ensure the best exploitation and exploration capabilities of the calculation.

The fitness function considers the number of features selected and the accuracy of the classifier. It reduces the size of features selected and increases the classification accuracy. Consequently, the subsequent fitness function is used to evaluate the individual solution.

(25) $$Fitness=\alpha \;*\;ErrorRate+\left(1-\alpha \right)\;*\;\frac{\#SF}{\#All\_F}$$

Here, α, which is usually set to 0.9, is used to control the importance of subset length and classification quality. ErrorRate is the classification error rate based on the number of features selected. ErrorRate can be evaluated as the percentage of incorrect classifications to the whole number of classifications made, and its values lie in the range of 0 to 1. ErrorRate is the complement of classification accuracy. #SF denotes the total features selected, and #All_F represents the overall number of features in the original data. 

### 3.4. Classification Model

For accurate classification of the intrusions, the LSTM-AE model is leveraged for both the identification and the classification of the intrusions. The deep RNN (particularly LSTM) model is the underlying structure of the DL model that is applied to time-series and sequential data to learn the features and patterns. But, the LSTM [21], out of the RNN method, contains memory cells for pattern recognition that is dependent on short -and long-term input datasets. These are beneficial in the detection and forecasting of the outliers in time-series datasets [22]. The LSTM cell comprises three memory gates such as the forget, input (update), and output gates.

(26) $$F_{t}=\delta (M_{f}(h_{t-1},x_{t})+B_{f})$$

In Equation (26), B and M denote the bias and weight of the LSTM, respectively. Furthermore, the dataset fed as input to the LSTM cells gets upgraded by the input gate $\left(I_{t}\right)$.

(27) $$l_{t}=\delta (M_{j}(h_{t-1)}x_{t})+B_{i})$$

(28) $${\bar{C}}_{t}=tanh(M_{c}(h_{t-1},x_{t})+B_{c})$$

At last, the output gate plays its role by transmitting the dataset created to the output cell $\left(H_{t}\right)$ or the succeeding state vector $\left(C_{t}\right)$.

(29) $$C_{t}=F_{t}⊗C_{t-1}+l_{t}$$

(30) $$H_{t}=\delta (M_{o}(h_{t-1)}x_{t})+B_{o})⊗tanh(C_{t})$$

In this expression, $x_{t}$ stands for input dataset, $h_{t-1}$ denotes the prior short-term state, and B and M represent the bias and weight matrices, correspondingly. Meanwhile, the LSTM model has a state vector $C_{t}$ that includes tanh and sigmoid functions. The model evaluates the gradient error at every time point, whereas the derivative items of tanh and sigmoid functions become additive. It prevents the model from suffering gradient disappearing problems. Unlike other gradient descent models, the LSTM exploits a mini-batch method at the time of training the data samples. Usually, the LSTM model comprises a single layer of cells, whereas the ensemble and a combination of numerous LSTM models increase the depth of the layer. This in turn increases the model’s performance and accuracy in training. It also helps in identifying the short- and long-term continuous patterns and big datasets.

On top of that, the AE method is utilized with LSTM for training the long-term patterns and the most important features. AE is an unsupervised type of ANN that intends to learn the essential hidden representation of the datasets by decoding and encoding processes. The output and the input datasets are compared to evaluate the differences. In the case of large differences, it shows that the reconstruction loss is higher. Based on this output, it can be assumed that the model can handle the reconstructed dataset. Accordingly, the data are recognized as irregular.

**Figure 2:**
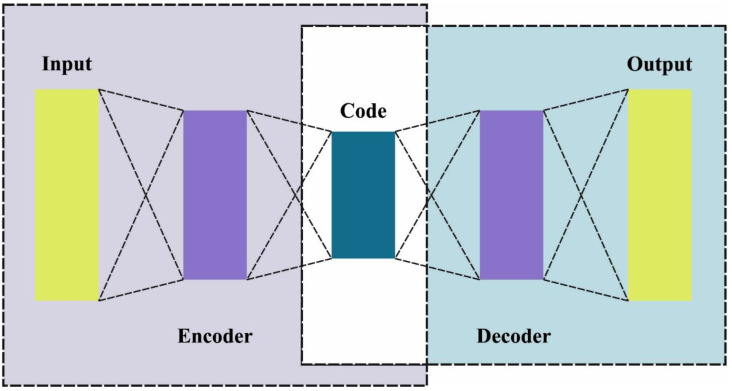
Structure of AE.

The LSTM-AE is an application of AE in which the LSTM cells are employed in the encoder–decoder layer. This setup brings the advantages of both methods for time-series or sequential datasets. In this work, the LSTM-AE is employed because it provides certain advantages over the normal (regular) AEs, for instance, LSTM-AE can handle sequence data as input (time-series dataset), whereas the normal AE cannot accept a sequential sample as the input dataset. Furthermore, the LSTM-AE models take a wider range of input lengths (short- or long-term), while on the other hand, the regular AE takes only a fixed size of the input dataset. At last, the data dimension increases, and the computation becomes complicated, since the long- and short-term dependence on time in previous data affects the current IIoT data. To resolve these problems, LSTM-AE is applied for the extraction of the fundamental feature with benefits over normal AEs. The structure of the AE is shown in Figure 2. 

The AE model comprises the output layer, input layer, and Hidden Layer (HL), whereas the interconnected layer is made up of an LSTM cell to create the output layer dataset. The HL takes the sample from various sampling times to estimate and calculate the impact on the succeeding sample datasets at another sampling period. The next time point values are attained as the output values by modeling and integrating the impact as the forecasted values. The respective sample of the HL is characterized by an equation in which the sample of $x_{i}$ remains the input instance of the data sample from X, $N_{w}$ signifies the weight matrices, and $P_{w}$ characterizes the bias vector between the input layer and the HLs. The function δ updates the next input layer to make $C_{i}$ as the output value in the AE architecture.

(31) $$C_{j}=\delta (N_{w}.x_{j}+P_{w})$$

### 3.5. Hyperparameter Tuning Model

At last, the RMSProp optimizer is exploited for the hyperparameter tuning of the LSTM-AE model. In the vertical direction, the RMSProp optimizer [22] restricts the oscillation. Thus, in the horizontal direction, the learning rate gets improved and the algorithm takes a large step in converging at a faster rate. The RMSProp calculation is given as follows. The value of the momentum is represented as beta and is set to 0.9 [22].

(32) $$v_{dw}=\beta \cdot v_{dw}+(1-\beta )\cdot dw^{2}$$

(33) $$v_{db}=\beta^{\prime }\cdot v_{dw}+(1-\beta )\cdot db^{2}$$

(34) $$W=W-\alpha \cdot \frac{dw}{\sqrt{v_{dw}}+\varepsilon }$$

(35) $$b=b-\alpha \cdot \frac{db}{\sqrt{vdb}+\varepsilon }$$

In backward propagation process, dW and *db* are used to update the W and b parameters with the help of the following expression [22]:

(36) W=W−learning rate ∗ dW

(37) b=b−learning rate ∗ db

Let us assume the exponentially-weighted average square values of dW and db instead of independently using the dW and *db* values for all the epochs [22].

(38) $$S_{dW}=\beta \;*\;S_{dW}+(1-\beta )\;*\;dW^{2}$$

(39) $$S_{db}=\beta \;*\;S_{db}+(1-\beta )\;*\;db^{2}$$

Here, β represents the hyperparameter in the range of 0 to 1. The newly weighed average can be formed with the help of current value square, weights and the average of the previous values. The parameters will be updated after the evaluation of the exponentially-weighted averages [22].

(40) W=W−learning rate ∗ dW/sqrt(S)

(41) b=b−learning rate ∗ db/sqrt(S)

$S_{dW}$ is relatively lower in such a way that it is divided by dW. Here, $S_{db}$ is relatively higher so that when it is divided by db with a comparatively large number, it slows down the update on the vertical dimension.

## 4. Results Analysis

The performance of the SCAVO-EAEID method was experimentally validated on two datasets such as NSL-KDD 2015 and CICIDS 2017. The proposed model was simulated in the Python 3.6.5 tool on a PC configured with these specifications; i5-8600k, GeForce 1050Ti 4GB, 16 GB RAM, 250 GB SSD, and 1 TB HDD. The parameter settings are given as follows: learning rate, 0.01; dropout, 0.5; batch size, 5; epoch count, 50; and activation, ReLU.

Table 1 reports the best cost outcomes of the proposed SCAVO-FS method and other FS algorithms on two datasets. The experimental values indicate that the proposed SCAVO-FS technique achieved the optimal best cost values such as 0.05101 and 0.41204 under the NSL-KDD-2015 and CICIDS-2017 datasets, correspondingly.

In Table 2, the FS results are represented in terms of several selected features. The results indicate the promising performance of the presented SCAVO-FS technique. Moreover, it is recognized that the SCAVO-FS technique effectually selected 41 and 80 features under NSL-KDD-2015 and CICIDS-2017 datasets, correspondingly.

**Figure 3:**
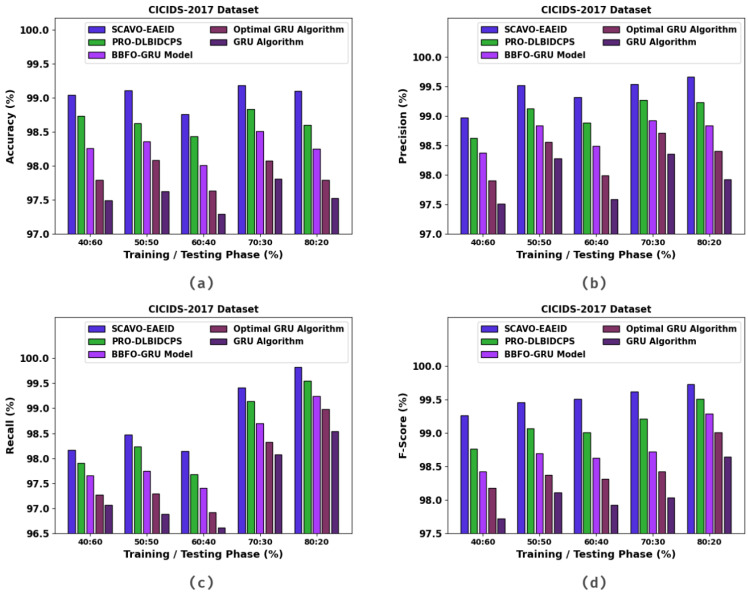
Overall classification outcomes of the proposed SCAVO-EAEID technique and other techniques on the NSL-KDD dataset.

In Table 3 and Figure 3, the experimental outcomes accomplished by the proposed SCAVO-EAEID method upon the NSL-KDD dataset are portrayed. The outcomes indicate that the SCAVO-EAEID technique achieved increased values under all the training set/testing set (TRS/TSS) instances. For instance, with 40:60 of TRS/TSS, the SCAVO-EAEID technique attained an $accu_{y}$ of 98.70%, $prec_{n}$ of 99.16%, $reca_{l}$ of 96.79%, and $F_{score}$ of 97.69%. Meanwhile, with 50:50 of TRS/TSS, the SCAVO-EAEID technique accomplished an $accu_{y}$ of 98.74%, $prec_{n}$ of 99.24%, $reca_{l}$ of 98.14%, and $F_{score}$ of 99.53%. Finally, with 80:20 of TRS/TSS, the SCAVO-EAEID technique acquired an $accu_{y}$ of 99.20%, $prec_{n}$ of 99.58%, $reca_{l}$ of 99.42%, and $F_{score}$ of 99.84%.

**Figure 4:**
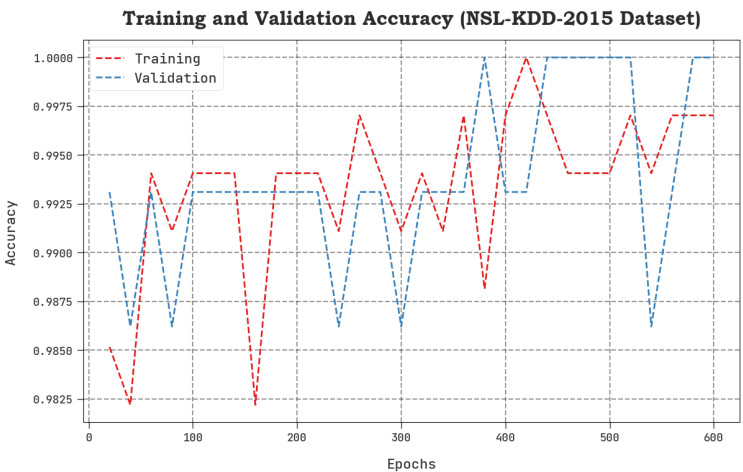
TACC and VACC analytical outcomes of the SCAVO-EAEID technique on the NSL-KDD dataset.

The TACC and VACC values, achieved by the proposed SCAVO-EAEID system upon the NSL-KDD 2015 dataset are shown in Figure 4. The outcomes exhibit that the SCAVO-EAEID method yielded better performance with maximum TACC and VACC values. It is noticeable that the SCAVO-EAEID methodology gained the maximum TACC outcomes.

**Figure 5:**
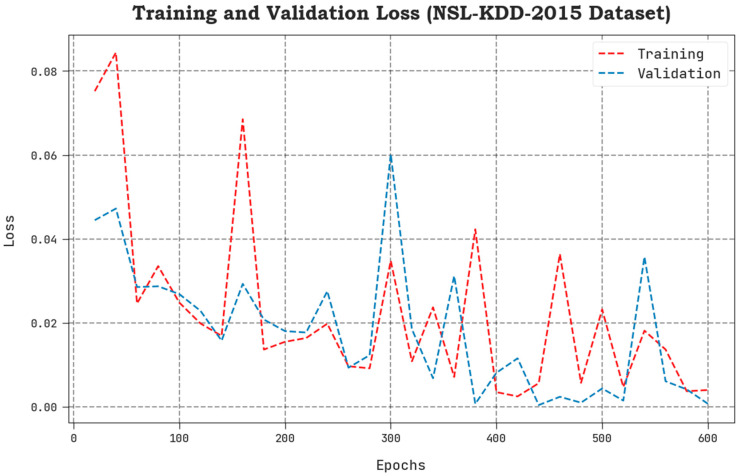
TLS and VLS analytical outcomes of the SCAVO-EAEID technique on the NSL-KDD dataset.

The TLS and VLS values, accomplished by the proposed SCAVO-EAEID system upon the NSL-KDD 2015 dataset, are shown in Figure 5. The results display that the SCAVO-EAEID approach showcased a superior performance with low TLS and VLS values. It is noticeable that the SCAVO-EAEID method achieved the least VLS outcomes.

**Figure 6:**
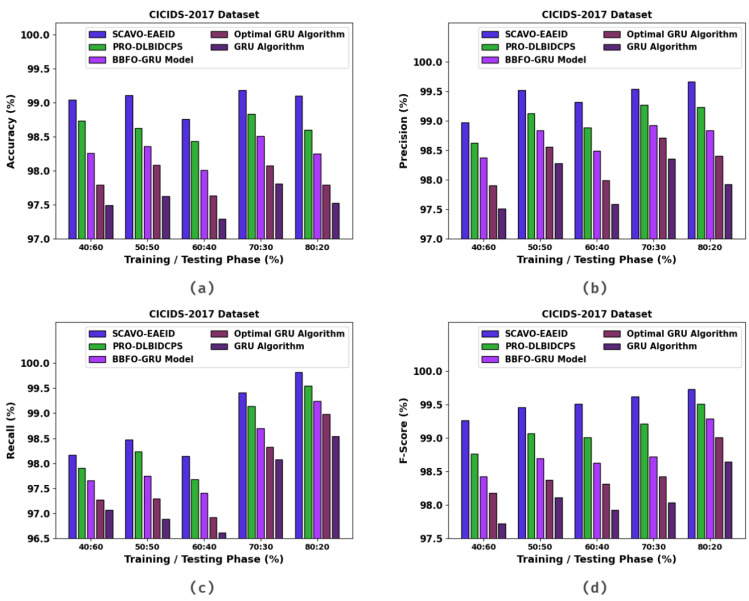
Overall classification outcomes of the SCAVO-EAEID and other techniques on the CICIDS-2017 dataset.

In Table 4 and Figure 6, the experimental outcomes of the SCAVO-EAEID method and other techniques upon the CICIDS-2017 dataset are portrayed. The outcomes indicate that the SCAVO-EAEID method achieved improved values under all the TRS/TSS instances. For instance, with 40:60 of TRS/TSS, the SCAVO-EAEID technique attained an $accu_{y}$ of 98.70%, $prec_{n}$ of 99.16%, $reca_{l}$ of 96.79%, and $F_{score}$ of 97.69%. Meanwhile, with 50:50 of TRS/TSS, the SCAVO-EAEID technique accomplished an $accu_{y}$ of 98.74%, $prec_{n}$ of 99.24%, $reca_{l}$ of 98.14%, and $F_{score}$ of 99.53%. Finally, with 80:20 of TRS/TSS, the SCAVO-EAEID method achieved an $accu_{y}$ of 99.10%, $prec_{n}$ of 97.92%, $reca_{l}$ of 98.54%, and $F_{score}$ of 98.64%.

**Figure 7:**
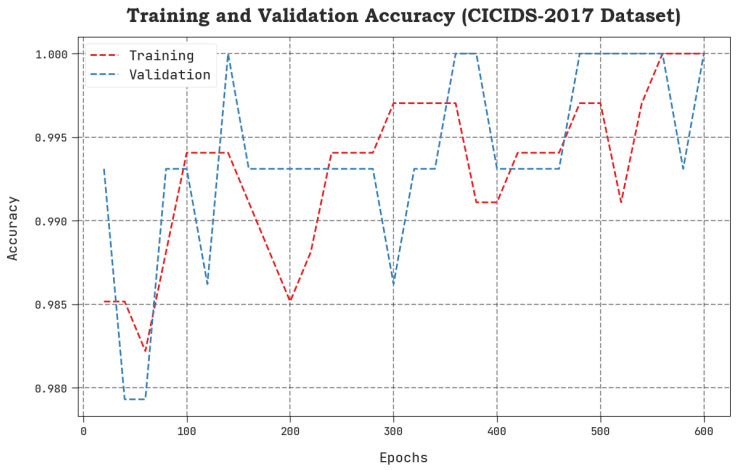
TACC and VACC analytical outcomes of the SCAVO-EAEID technique upon the CICIDS-2017 dataset.

The TACC and VACC values, achieved by the proposed SCAVO-EAEID method upon the CICIDS-2017 dataset are shown in Figure 7. The outcomes demonstrate that the SCAVO-EAEID method achieved a superior performance with maximum TACC and VACC values. Notably, the SCAVO-EAEID methodology attained the highest TACC outcomes.

**Figure 8:**
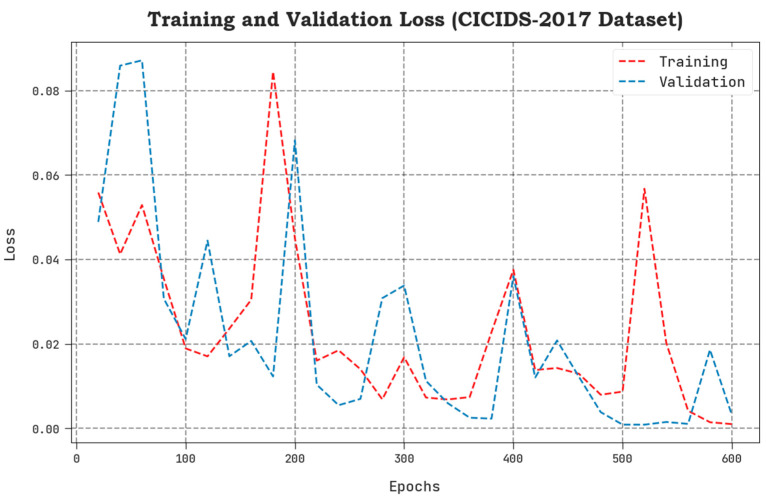
TLS and VLS analytical outcomes of the SCAVO-EAEID method upon the CICIDS-2017 dataset.

The TLS and VLS values, acquired by the proposed SCAVO-EAEID technique upon the CICIDS-2017 dataset, are portrayed in Figure 8. The results confirm that the SCAVO-EAEID method achieved a superior performance with low TLS and VLS values. Notably, the proposed SCAVO-EAEID method displayed the minimal VLS outcomes.

**Figure 9:**
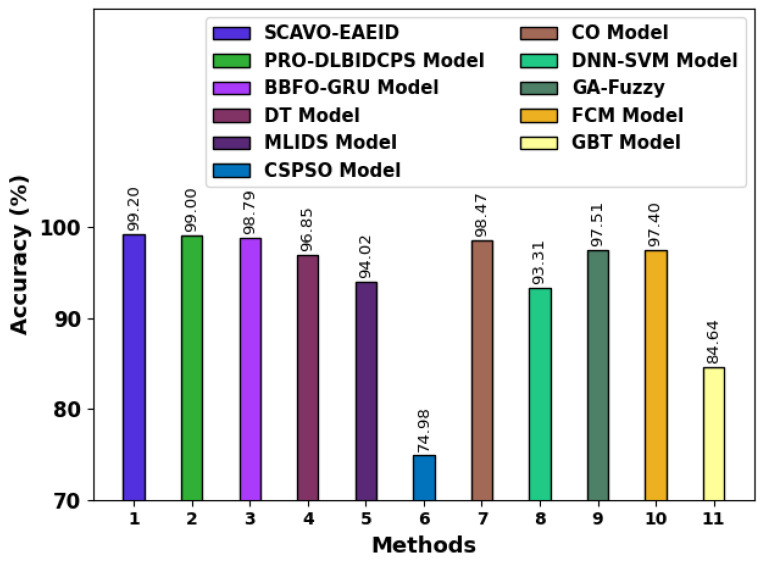
Overall $accu_{y}$ outcomes of the SCAVO-EAEID and other recent techniques.

A comparative $accu_{y}$ examination was conducted between the proposed SCAVO-EAEID technique and other recent methods [12,23] and the results are shown in Table 5 and Figure 9. The outcomes infer that the SCAVO-EAEID technique accomplished the maximum $accu_{y}$ of 99.20%. Contrastingly, the rest of the models such as PRO-DLBIDCPS, BBFO-GRU, DT Model, MLIDS Model, CSPSO Model, CO Model, DNN-SVM Model, GA-Fuzzy, FCM Model, and GBT Model attained minimum $accu_{y}$ values such as 99.00%, 98.79%, 96.85%, 94.02%, 74.98%, 98.47%, 93.31%, 97.51%, 97.4%, and 84.64%, respectively.

To exhibit the enhanced performance of the SCAVO-EAEID technique, a brief time complexity analysis was conducted and the results are shown in Table 6. The outcomes infer that the DNN-SVM, GA-Fuzzy, FCM Model, GBT Model, BBFO-GRU, MLIDS, and CSPSO techniques demanded higher TRT and TST values. However, the SCAVO-EAEID technique accomplished a superior performance with minimal TRT and TST values such as 0.542 min and 0.246 min, respectively. These results highlight the supremacy of the proposed SCAVO-EAEID technique.

## 5. Conclusions

In this study, a new SCAVO-EAEID system has been introduced for intrusion classification in the CPS environment. The presented SCAVO-EAEID method emphasizes the detection of intrusions in the CPS environment using major sub-processes, namely, LSTM-AE-based classification, Z-score normalization, the SCAVO-FS technique, and RMSProp optimizer-based hyperparameter tuning. At the primary level, the SCAVO-EAEID technique applies Z-score normalization as a preprocessing step. Moreover, the SCAVO-FS technique is derived to elect the optimal feature subsets. Furthermore, the LSTM-AE model is applied for the detection and classification of intrusions. At last, the RMSProp optimizer is used for hyperparameter tuning of the LSTM-AE model. To demonstrate the remarkable performance of the proposed SCAVO-EAEID technique, two benchmark datasets were used. The experimental outcomes reiterated the significant performance of the proposed SCAVO-EAEID technique over other approaches. In the future, the performance of the presented method can be enhanced using metaheuristics-based feature selection techniques. 

## Figures and Tables

**Table 1:** Best cost results of the SCAVO-FS technique and other techniques.

Best Cost		
Methods	NSL-KDD-2015	CICIDS-2017
SCAVO-FS	0.05101	0.41204
AHSA-FS	0.05433	0.04311
BBFO-FS	0.07382	0.06445
BFO-FS	0.09371	0.08753
SSO-FS	0.10384	0.09422
WOA-FS	0.11940	0.11790

**Table 2:** FS Results of the proposed SCAVO-FS technique and other techniques.

Number of Selected Features		
Methods	NSL-KDD-2015	CICIDS-2017
Total Features	41	80
SCAVO-FS	14	17
AHSA-FS	15	19
BBFO-FS	18	24
BFO-FS	19	30
SSO-FS	20	28
WOA-FS	20	33

**Table 3:** Classification outcomes of the proposed SCAVO-EAEID technique and other techniques on the NSL-KDD dataset.

Training/Testing Phase (%)	Accuracy	Precision	Recall	F-Score
40:60				
SCAVO-EAEID	98.70	99.16	98.13	99.23
PRO-DLBIDCPS	98.29	98.80	97.74	98.91
BBFO-GRU Model	97.92	98.44	97.42	98.41
Optimal GRU Algorithm	97.44	98.21	97.02	98.05
GRU Algorithm	97.16	97.85	96.79	97.69
50:50				
SCAVO-EAEID	98.74	99.24	98.14	99.53
PRO-DLBIDCPS	98.48	99.03	97.92	99.30
BBFO-GRU Model	98.12	98.73	97.65	98.96
Optimal GRU Algorithm	97.92	98.32	97.27	98.53
GRU Algorithm	97.63	97.87	96.80	98.27
60:40				
SCAVO-EAEID	98.91	99.50	98.17	99.71
PRO-DLBIDCPS	98.41	99.15	97.90	99.30
BBFO-GRU Model	97.96	98.71	97.54	98.87
Optimal GRU Algorithm	97.62	98.34	97.21	98.60
GRU Algorithm	97.25	97.99	96.86	98.40
70:30				
SCAVO-EAEID	98.95	99.50	99.12	99.81
PRO-DLBIDCPS	98.6	99.15	98.81	99.58
BBFO-GRU Model	98.33	98.93	98.45	99.19
Optimal GRU Algorithm	98.02	98.44	97.99	98.69
GRU Algorithm	97.69	98.16	97.62	98.29
80:20				
SCAVO-EAEID	99.20	99.58	99.42	99.84
PRO-DLBIDCPS	99.00	99.12	99.03	99.41
BBFO-GRU Model	98.79	98.89	98.55	98.95
Optimal GRU Algorithm	98.49	98.47	98.24	98.52
GRU Algorithm	98.24	98.16	97.91	98.26

**Table 4:** Classification outcomes of the SCAVO-EAEID and other techniques on the CICIDS-2017 dataset.

Training/Testing Phase (%)	Accuracy	Precision	Recall	F-Score
40:60				
SCAVO-EAEID	99.04	98.97	98.17	99.26
PRO-DLBIDCPS	98.73	98.63	97.91	98.76
BBFO-GRU Model	98.26	98.38	97.65	98.42
Optimal GRU Algorithm	97.79	97.90	97.27	98.18
GRU Algorithm	97.49	97.51	97.07	97.72
50:50				
SCAVO-EAEID	99.11	99.52	98.47	99.46
PRO-DLBIDCPS	98.62	99.13	98.23	99.07
BBFO-GRU Model	98.36	98.84	97.74	98.69
Optimal GRU Algorithm	98.08	98.56	97.29	98.37
GRU Algorithm	97.62	98.28	96.88	98.11
60:40				
SCAVO-EAEID	98.76	99.32	98.14	99.51
PRO-DLBIDCPS	98.43	98.89	97.68	99.01
BBFO-GRU Model	98.01	98.49	97.40	98.63
Optimal GRU Algorithm	97.63	97.99	96.92	98.31
GRU Algorithm	97.29	97.59	96.61	97.92
70:30				
SCAVO-EAEID	99.18	99.54	99.42	99.62
PRO-DLBIDCPS	98.83	99.27	99.14	99.21
BBFO-GRU Model	98.51	98.93	98.70	98.72
Optimal GRU Algorithm	98.07	98.71	98.33	98.42
GRU Algorithm	97.81	98.36	98.07	98.03
80:20				
SCAVO-EAEID	99.10	99.67	99.82	99.73
PRO-DLBIDCPS	98.60	99.23	99.55	99.51
BBFO-GRU Model	98.25	98.84	99.24	99.29
Optimal GRU Algorithm	97.79	98.40	98.98	99.01
GRU Algorithm	97.52	97.92	98.54	98.64

**Table 5:** Comparative $accu_{y}$ analysis outcomes achieved by the proposed SCAVO-EAEID technique and other techniques.

Methods	Accuracy (%)
SCAVO-EAEID	99.20
PRO-DLBIDCPS Model [12]	99.00
BBFO-GRU Model [23]	98.79
DT Model [12]	96.85
MLIDS Model [12]	94.02
CSPSO Model [12]	74.98
CO Model [12]	98.47
DNN-SVM Model [12]	93.31
GA-Fuzzy [12]	97.51
FCM Model [12]	97.4
GBT Model [12]	84.64

**Table 6:** Time complexity analysis outcomes of the SCAVO-EAEID and other techniques.

Methods	Training Time (min)	Testing Time (min)
SCAVO-EAEID	0.542	0.246
PRO-DLBIDCPS Model [12]	0.752	0.381
BBFO-GRU Model [23]	1.106	0.363
DT Model [12]	0.888	0.677
MLIDS Model [12]	1.212	0.331
CSPSO Model [12]	1.242	0.425
CO Model [12]	0.802	0.572
DNN-SVM Model [12]	1.384	0.996
GA-Fuzzy [12]	1.351	0.444
FCM Model [12]	1.749	0.873
GBT Model [12]	1.463	0.875

## Data Availability

Data sharing not applicable to this article as no datasets were generated during the current study.
